# Untangling Mephedrone‐Induced Social Reward in Rats—The Involvement of Oxytocin and Vasopressin V_1A_ Receptors

**DOI:** 10.1111/gbb.70057

**Published:** 2026-07-10

**Authors:** Olga Wronikowska‐Denysiuk, Łukasz Kurach, Radosław Mlak, Dominika Przygodzka, Tymoteusz Słowik, Barbara Budzyńska

**Affiliations:** ^1^ Independent Laboratory of Behavioral Studies, Chair of Biomedical Sciences Medical University of Lublin Lublin Poland; ^2^ Department of Laboratory Diagnostics Medical University of Lublin Lublin Poland; ^3^ Chair and Department of Forensic Medicine Medical University of Lublin Lublin Poland; ^4^ Experimental Medicine Center Medical University of Lublin Lublin Poland

## Abstract

The rewarding effects of psychostimulants are strongly influenced by social context, yet the underlying neurochemical and neuropeptidergic mechanisms remain unclear. This study examined the role of oxytocin (OT) and vasopressin (AVP) in context‐dependent reward induced by subchronic mephedrone treatment in rats. Using classic‐ and social‐conditioned place preference (CPP) we assessed reward and later—monoamine levels in the prefrontal cortex (PFC) and hippocampus, plasma OT and AVP levels, and mRNA expression of oxytocin (OTRs) and vasopressin V1A (AVPV_1A_Rs) receptors in the nucleus accumbens (NAc) and hippocampus. Pharmacological manipulations of OTRs and AVPV_1A_Rs were used to evaluate their role in social‐CPP. We confirmed that mephedrone (5 mg/kg) induced social‐ but not classic‐CPP. In classic‐CPP, mephedrone elevated PFC dopamine, an effect abolished under social conditioning, which instead reduced hippocampal serotonin and noradrenaline. Social context alone lowered baseline monoamines, suggesting dampened neurochemical signaling. Social conditioning increased plasma OT and AVP, whereas mephedrone selectively reversed the OT rise. At the receptor level, mephedrone up‐regulated AVPV_1A_R mRNA in the hippocampus and OTR mRNA in the NAc, indicating central adaptations distinct from peripheral peptide changes. Behaviorally, social‐CPP was abolished by OTR agonism (carbetocin) or AVPV_1A_R antagonism (SR49059), while OTR antagonism (L‐368,899) or peripheral AVP had no effect. These findings show that mephedrone‐induced social reward is shaped by OT and AVP systems. Social context modulates peripheral neuropeptides and engages central receptor adaptations in the hippocampus and NAc, influencing behavioral outcomes. This study provides insights into the neurobiological mechanisms through which the social environment alters drug reward.

## Graphical abstract

Social context alters the neurochemical and neuropeptidergic mechanisms of mephedrone reward, revealing a dual regulation by monoamines and oxytocin/vasopressin signaling in brain regions critical for reinforcement.

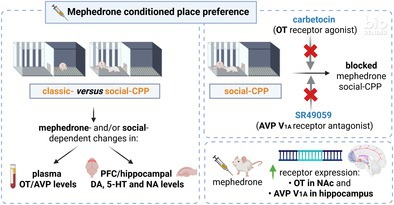

## 1 Introduction

According to the newest analysis published in 2025 by the European Union Drug Agency (EUDA), the European Union is facing emerging addiction‐related problems. Specifically, the most concerning risks include the high availability of a wider range of often more potent substances, drug‐related violence, as well as polydrug use and the mis‐selling of drugs (EUDA: [1]). Susceptibility to addiction to psychoactive substances is shaped by a range of factors, including genetic predisposition, co‐occurring mental health disorders, environmental conditions, peer influence, drug availability, and neighborhood characteristics [2, 3]. Notably, the social context in which drug use occurs has been implicated in both the onset and progression of addictive behaviors [4], and is consistently associated with heightened vulnerability to substance misuse [5]. The interplay between the above‐mentioned environmental influences and addiction risk in humans is being increasingly explored at the molecular level using animal models. A substantial amount of animal research has demonstrated that the presence of a social companion during drug exposure can significantly alter the magnitude of drug‐induced reward [6, 7, 8, 9]. In our previous studies, we also observed a similar phenomenon and a social‐dependent response to mephedrone (4‐methylmethcathinone) exposure in rats [10].

Mephedrone is a primary representative of synthetic cathinones, which are increasingly produced in Europe and more often used as substitutes for traditional stimulants, like amphetamine [11]. Furthermore, the EU Early Warning System discovered that synthetic cathinones were more frequently mis‐sold as MDMA or used to adulterate MDMA. The mechanism of action of mephedrone has been extensively studied, demonstrating its ability to modulate the levels of specific monoamines within the central nervous system (CNS). It enhances extracellular concentrations of dopamine (DA), serotonin (5‐HT), and noradrenaline (NA) by inhibiting their reuptake through interactions with plasma membrane monoamine transporters [12, 13, 14, 15, 16, 17].

Nevertheless, the molecular mechanisms underlying the influence of social context on the action of mephedrone remain poorly understood. Prior investigations conducted in our laboratory revealed that mephedrone can induce a social‐ and dose‐dependent response in the conditioned place preference (CPP) paradigm. Specifically, a low dose of mephedrone—which does not produce place preference in the classic‐CPP procedure—elicited a significant place preference when administered in a social‐CPP setting, i.e., when an identically treated conspecific was present during the conditioning phase, suggesting a rewarding effect modulated by social interaction [10]. Motivated by this phenomenon, we aimed to explore the molecular mechanisms underlying the observed effects. Literature indicates that oxytocin (OT) and arginine‐vasopressin (AVP) play crucial roles as modulators of the addictive properties of psychoactive substances, affecting both social‐context‐dependent and independent addiction processes (for further details, see [18]).

OT and AVP, two neurohormones produced in the hypothalamus, are involved in regulating a wide array of physiological processes. OT plays a critical role in childbirth, milk production, and mother–child bonding, while also influencing romantic relationships, sexual reproduction, emotional regulation, and social cognition, such as trust and empathy [19, 20, 21]. On the other hand, AVP, also referred to as antidiuretic hormone, primarily functions to regulate blood pressure through peripheral vasoconstriction and by enhancing water reabsorption in the kidneys [22]. Despite these distinct roles, both OT and AVP share a common involvement in regulating social behavior, emotional responses, and stress reactions [23]. Given their profound influence on social and emotional processes, there is increasing interest in targeting the OT and AVP systems as potential therapeutic strategies for addressing substance abuse. Briefly, it has been shown that activation of OT transmission is linked to the reduction of the rewarding effects of various drugs of abuse. In contrast, the effects of AVP ligands on drug reward are receptor type‐dependent, as detailed in the discussion section. These findings suggest that targeting OT and AVP systems could modulate drug reward and addiction‐related behaviors [18].

It is essential to emphasize that mephedrone is frequently compared to MDMA due to their similarities in psychoactive effects, as both substances elicit euphoria, enhanced sociability, and heightened sensory perception [24]. However, mephedrone demonstrates a higher abuse potential compared to MDMA. Its faster onset and shorter duration of effects, likely due to its brief elimination half‐life, may contribute to a more compulsive pattern of use. Furthermore, mephedrone is characterized by a more pronounced stimulant effect, whereas MDMA is primarily associated with entactogenic properties [25, 26]. This distinction is also reflected in animal research on the effects of OT and AVP in drug abuse, which highlights drug‐dependent effects, with different responses observed for classic empathogens like MDMA versus other psychoactive substances that exert more stimulant effects. MDMA induces prosocial effects such as increased social bonding, similar to OT and AVP actions [7, 27] and has been shown to elevate plasma OT levels in both animals [28] and humans [29]. In contrast, chronic use of non‐empathogenic drugs like morphine, cocaine, and ethanol reduces OT levels [30, 31, 32]. Additionally, studies in rats using lever drug discrimination paradigms (MDMA vs amphetamine) indicate that substitution with OT receptor (OTR) agonists, like carbetocin, enhances MDMA‐seeking behavior, while OTR antagonist (atosiban) disrupts MDMA‐ but not amphetamine‐lever responding [33]. These findings suggest that OT/AVP effects may vary between empathogens and classic psychostimulants.

Based on the ability and potency of drugs to inhibit monoamine transporters, mephedrone is classified as lying between empathogens (MDMA) and classic stimulants (like cocaine) [34]. Consequently, a deeper understanding of the effects of OT and AVP ligands could provide valuable insights into the modulation of the reinforcing and addictive properties of mephedrone, as well as other substances with analogous pharmacodynamic profiles, thereby contributing to the development of more targeted therapeutic interventions for substance use disorders.

Therefore, the objective of the presented study was to evaluate the involvement of OT and AVP systems in mephedrone social‐reward. Specifically, the impact of OTRs and AVP receptors (AVPRs) ligands on the expression of mephedrone reward was assessed in the social‐CPP paradigm. This was complemented with the determination of mephedrone‐dependent changes in plasma OT and AVP levels, and in the prefrontal cortex (PFC) and hippocampal DA, 5‐HT, and NA levels. Finally, mephedrone‐induced changes in OTRs and AVPRs gene expression were analyzed in the hippocampus and in the nucleus accumbens (NAc). Altogether, the presented study provides a valuable contribution to understanding the complex interplay between the OT/AVP systems and the effects of mephedrone, with particular emphasis on the influence of social factors.

## 2 Materials and Methods

### 2.1 Animals

Male Wistar rats ( *Rattus norvegicus* , strain: Wistar, RRID:RGD_2312511) were obtained from the Centre of Experimental Medicine at the Medical University of Lublin (Poland), where all the experiments were conducted. At the beginning of the experiments, rats were approximately 8 weeks old and weighed 200–250 g. The animals were housed and cared for under standard laboratory conditions, which included: (1) a 12‐h light/dark cycle with lights on at 8:00 am (experiments were conducted during the light phase between 8.30 am—5.30 pm); (b) a room temperature of 21°C ± 1°C; and (c) a relative humidity of 50% ± 5%. Throughout the duration of the study, except during testing periods, the animals had *ad libitum* access to laboratory chow (Agropol, Poland) and water. Animals were housed in pairs with a weight‐similar conspecific that was receiving the same treatment but, importantly, cage‐mates were not social companions to each other during social‐CPP. Each animal during social‐CPP was paired with an identically treated conspecific that was not their cage‐mate.

### 2.2 Ethics Statement

All experiments: (1) adhered to the ARRIVE guidelines; (2) were conducted in accordance with the National Institutes of Health Guidelines for the Care and Use of Laboratory Animals, as well as the European Community Council Directive for the Care and Use of Laboratory Animals of September 22, 2010 (2010/63/EU); and (3) received approval from the Local Ethics Committee in Lublin, Poland (Permissions No: 59/2021 and 52/2022).

### 2.3 Drugs

*Mephedrone hydrochloride* (Tocris Bioscience, Cat. No. 4443) was administered subchronically: (a) in the classic‐ and social‐CPP protocol: once a day, for 6 days of conditioning, during the afternoon session and (b) for the determination of OTRs and AVPRs gene expression (analogously as in the CPP protocol): once a day, for 6 days. Mephedrone was administered at the dose of 5 mg/kg based on our previous studies that showed effectiveness of this dose in the social‐CPP paradigm [10]. *Carbetocin* (long lasting oxytocin analog that displays agonist properties, Tocris Biotechne Cat. No. 4852), *L‐368,899 hydrochloride* (non‐peptide OTR antagonist, Tocris Biotechne Cat. No. 2641), *AVP* (non‐selective AVPRs agonist, Tocris Biotechne Cat. No. 2935) or *SR49059* (selective non‐peptide AVPV_1A_R antagonist, Sigma‐Aldrich Cat. No. S5701) were administered acutely on the last day of social‐CPP experiment prior to the post‐conditioning test. To ensure that the selected compounds would be effective during the post‐conditioning test, the doses and timing of treatment were determined based on a thorough literature review and adjusted after our pilot studies. This review took into account the effectiveness of the tested doses within the given time frame and was adjusted to match the duration of the post‐conditioning test (15 min). Therefore, *carbetocin* was administered at the doses of 3.2 and 6.4 mg/kg 15 min prior to the post‐conditioning test [30, 32, 35, 36], *L‐368,899* was administered at the doses of 2.5 and 5 mg/kg 45 min prior to the post‐conditioning test [37, 38, 39, 40], *AVP* was administered at the doses of 0.01 and 0.0025 mg/kg 20 min prior to the post‐conditioning test [27] and *SR49059* was administered at the doses of 0.25 and 0.5 mg/kg 30 min prior to the post‐conditioning test [27, 41]. All drug solutions were prepared according to the manufacturer's solubility data. Mephedrone hydrochloride, carbetocin and AVP were dissolved in 0.9% sterile saline, L‐368,899 hydrochloride was suspended in the 0.9% sterile saline with the drop of Tween 80 (not exceeding a concentration of 0.25% Tween 80) and SR49059 was firstly dissolved in the DMSO (final concentration of 1%) and then suspended in the 0.9% sterile saline with the drop of Tween 80 (not exceeding a concentration of 0.25% Tween 80). All drugs were administered intraperitoneally (ip) at a volume of 2 mL/kg. Control groups received either 0.9% sterile saline or the corresponding vehicle (ip, 2 mL/kg).

### 2.4 CPP Apparatus and Software

The CPP test has been repeatedly performed and the chosen setup was validated in our laboratory [10, 42, 43]. In brief, the CPP test was carried out in Ugo Basile two‐compartment CPP boxes, separated by guillotine doors. Each compartment measured 30 × 30 × 30 cm. The two compartments differed in wall colors/patterns and floor grids: one had black walls with square 0.1 × 0.1 cm holes in the floor, while the other had black and white striped walls with round 0.2 cm holes in the floor. Both the pre‐ and post‐conditioning sessions were recorded, and Ethovision XT 15 software (Noldus Information Technology, Wageningen, The Netherlands) was used to track the animals' locations in each compartment, based on the contrast between the animal and the floor. This software facilitated the measurement of time spent in each compartment, as well as the measurements of the distance traveled by each animal.

### 2.5 Classic‐ and Social‐CPP Protocol

**FIGURE 1:**
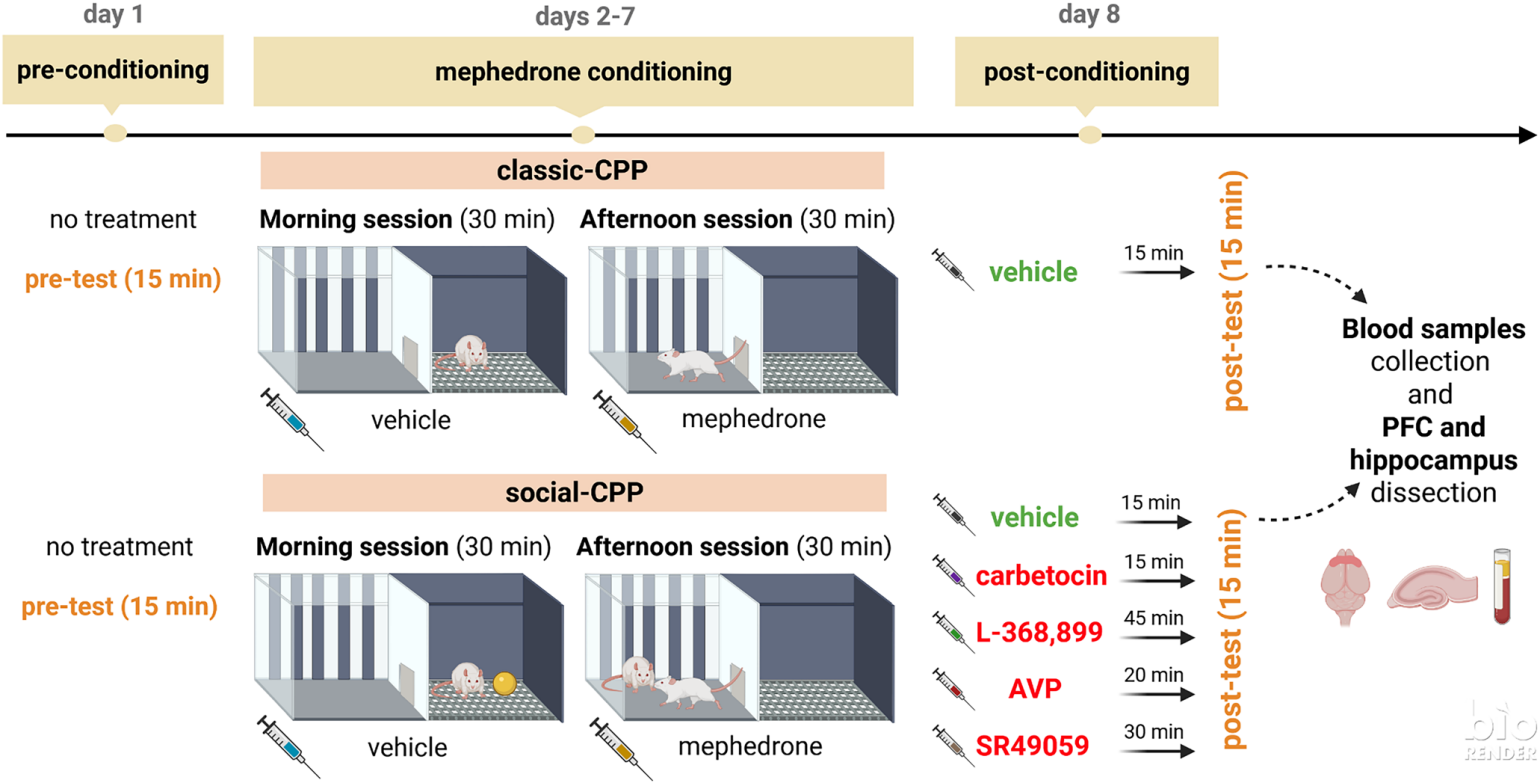
Experimental protocol for the classic‐ and social‐CPP test.

In the presented paper, the CPP paradigm was performed in two versions (classic‐ and social‐CPP, that differed in the conditioning phase) and was conducted as we previously described [10, 42, 43]. The phases of the CPP test consisted of: (1) *handling—*animals were handled by the experimentator every other day during a 7‐day time period in order to minimize the stress of the animals; (2) *habituation* (Day 0)—animals were individually put in the CPP boxes for 15 min for habituation in order to avoid flawed measurements during the pre‐conditioning test due to the stressor of an unknown environment; (3) *pre‐conditioning test* (Day 1)—during this phase animals did not receive any treatment; the guillotine doors were open and rats were able to freely explore the apparatus for 15 min during which initial preference was measured; (4) *classic‐ or social‐conditioning*‐—(Days 2–7) the conditioning phase was divided into 2 sessions per day (morning and afternoon session) separated by a minimum 4 h time period for each animal. The guillotine doors were closed. During the morning session, animals received vehicle injection and were individually put into one of the compartments alone *(classic‐CPP)* or with a non‐social object, a yellow tennis ball *(social‐CPP)* for 30 min. During the afternoon session, animals received an injection of mephedrone 5 mg/kg, a dose that we have previously shown to be ineffective in classic‐CPP and to have a rewarding effect in social‐CPP [10]. After the injection, rats were immediately put in the other compartment for 30 min alone *(classic‐CPP)* or with an identically treated, previously non‐familiar conspecific *(social‐CPP)*. The same partner rat was used for each conditioning session throughout the conditioning phase and, in order to adhere to the 3R rule, both rats at the same time served as “tested rats” and “partner rats”. In both versions of the test, animals were randomized within each experimental group, with half being conditioned in the “black” compartment and the other half in the “striped” compartment; (5) *post‐conditioning test* (Day 8)—on the last day of the experiment, animals receive vehicle injection *(classic‐CPP)* or were acutely injected vehicle or with OTRs and AVPRs ligands *(social‐CPP)* and then tested for post‐conditioning preference for 15 min with guillotine doors opened. Precisely, (a) carbetocin (OTRs agonist) at the doses of 3.2 and 6.4 mg/kg was injected 15 min prior to the post‐conditioning test; (b) L‐368,899 (OTRs antagonist) at the doses of 2.5 and 5 mg/kg was injected 45 min prior to the post‐conditioning test; (c) AVP (AVPRs agonist) the doses of 0.01 and 0.0025 mg/kg was injected 20 min prior to the post‐conditioning test and (d) SR49059 (AVPV_1A_R antagonist) at the doses of 0.25 and 0.5 mg/kg was injected 30 min prior to the post‐conditioning test. The rationale for the chosen doses and treatment‐to‐testing regimen was described in detail above in the *Drugs section*. After the post‐conditioning test, animals were decapitated and blood samples were collected for further ELISA analysis (described in detail below). The experimental protocol for the classic‐ and social‐CPP test has been presented in Figure 1.

### 2.6 Locomotor Activity

To assess whether the administered drugs affected the animals' mobility during the CPP test, the distance traveled by each animal was recorded over a 15‐min period during the post‐conditioning test. As with the CPP test, Ethovision XT 15 software was used for this analysis.

### 2.7 Evaluation of PFC and Hippocampal Neurotransmitters' (DA, 5‐HT and NA) Level Post Mephedrone Classic‐ or Social‐Conditioning

Tissue samples were mechanically homogenized in methanol by zirconium beads 1.5 mm (TriplePure, Benchmark) and BeadBug6 Homogenizer (Benchmark). Homogenate was centrifuged at 13,000 rpm, 10 min., at +4°C. 20 μL of supernatant was spiked with 10 μL of IS solution (500 ng/mL of dopamine‐d4). A 10 μL of aliquot was injected into the LC–MS/MS system for analysis. The quantification of DA, NA and 5‐HT was performed using high‐performance liquid chromatography (HPLC 1260 Agilent Technologies, Germany) coupled with a triple quadrupole mass spectrometer (QqQ 6460, Agilent Technologies, USA). Chromatographic separation was achieved on a thermostated (40°C) column (ReproSil Gold 120 C18 100 × 2 mm; 3 μm; Dr. Maisch, Germany) with elution A: water with the addition of 0.1% formic acid and B: acetonitrile in gradient as follows: B: 0–2.8 min.–5%; 3–4 min.–95%; 17 min.–95% with a flow rate of 0.2 mL/min. Electrospray ionization (ESI) was employed as the ion source, with operational parameters outlined in Table 1. Selected reaction monitoring (MRM) was applied for qualitative and quantitative analysis, with ESI‐MS/MS parameters and retention times provided in Table 2. Data acquisition and processing were carried out using MassHunter 10.0 software.

**TABLE 1:** Source parameters (ESI).

Gas temp.	325°C
Vaporizer	350°C
Gas flow	5 L/min.
Nebulizer	20 psi
Capillary	4500 V positive
Corona current	4 μA

**TABLE 2:** Parameters and retention time for selected compounds.

Compound name	Precursor ion (*m*/*z*)	Product ion (*m*/*z*)	Fragmentor	Collision energy (V)	Retention time (min)
DA	154	137 91	60	4 36	1.44
5‐HT	177	160 115	60	4 28	1.60
NA	170	115 152	60	4 20	1.40
DA‐D4	158	141 95	60	8 24	1.44

### 2.8 ELISA Analysis of OT and AVP Plasma Level Changes After Mephedrone Conditioning in Classic‐ and Social‐CPP

For solid‐phase extraction (SPE), samples were diluted 1:2 with 0.1% trifluoroacetic acid (TFA) and centrifuged at 15,000 RPM for 15 min. The extraction was performed using HyperSep C18 cartridges (200 mg, Thermo Scientific), preconditioned sequentially with 1 mL of acetonitrile (ACN) followed by 1 mL of 0.1% TFA in water. After sample loading, the cartridges were washed with 6 mL of 0.1% TFA in water, and analytes were eluted with 2 mL of 95% ACN containing 0.1% TFA. The eluted samples were evaporated to dryness under a steady stream of air at 37°C and subsequently reconstituted in sample buffer diluent [44]. Oxytocin and vasopressin concentrations were measured using commercially available kits from Wuhan EIAab Science Co., following the manufacturer's protocol.

### 2.9 Assessment of Mephedrone‐Induced Changes in OTRs and AVPRs Gene Expression in the Hippocampus and in the NAc

After 6 days of treatment (24 h after the last mephedrone injection), rats were decapitated and the hippocampus and NAc were dissected and preserved in the RNA later stabilisation solution (Invitrogen by Thermo Fisher Scientific). NAc was bilaterally dissected along with the centrally located portion of the anterior commissure. Coordinates were determined according to the Paxinos and Watson atlas (6th Edition): anteroposterior (AP): +1.89 to +1.14 mm from bregma; mediolateral (ML): +1 to +7.5 mm; dorsoventral (DV): −6.3 to −7.5 mm from the skull surface. The mRNA isolation from tissue samples was conducted using a column‐based method with a dedicated kit (RNeasy Plus Universal Mini Kit, Qiagen, Hilden, Germany) following the manufacturer's protocol. The purified mRNA samples were then reverse transcribed into complementary DNA (cDNA) in a thermocycler (T‐Personal 48, Biometra GmbH—Analytik Jena GmbH+Co. Jena, Germany) using a dedicated kit (cDNA Reverse Transcription Kit, Thermo Fisher Scientific, Waltham, MA, USA) following the manufacturer's protocol. The amplification was carried out on a StepOnePlus device (Applied Biosystems, Foster City, CA, USA) following the manufacturer's instructions. For the real‐time PCR, 96‐well plates were employed, and each well had a total reaction volume of 20 μL. This included 10 μL of TaqMan Universal Master Mix II, with UNG (Thermo Fisher Scientific, USA), 1 μL of TaqMan Gene Expression Assays (Thermo Fisher Scientific, USA) specifying Oxt receptor (OxtR, Assay ID: Rn00563503 m1); Avp receptor 1A (Avpr1a, Assay ID: Rn00583910 m1); Avp receptor 1B (Avpr1b, Assay ID: Rn01490541 m1), 4 μL of RNase‐free water, and 5 μL of diluted (1:10) cDNA template. Each sample was analyzed in triplicate. Relative expression of tested genes was normalized to GADPH (Assay ID: Rn01775763_g1, Thermo Fisher Scientific, USA) as a reference using 2^−∆∆Ct^ and 2^−∆Ct^ formulae.

### 2.10 Statistical Analysis

Statistical analysis and graph creation were performed using GraphPad Prism (version 8.0.1). Firstly, data underwent normal distribution analysis with the D'Agostino and Pearson tests. After confirming normal distribution of data, appropriate parametric tests were used. For the analysis of the classic‐ and social‐CPP, locomotor activity and OT/AVP plasma level, two‐way analysis of variance (ANOVA) with multiple comparisons with a post hoc Tukey's test was used. The mephedrone‐induced changes in OTRs and AVPRs in the hippocampus and in the NAc were analyzed with an unpaired, two‐tailed *t* ‐test (vehicle vs mephedrone for each receptor in each brain region). Data have been visualized on the graphs as individual values and as means of scores ± standard deviation (SD). Precisely, data have been shown: (1) in classic‐ and social‐CPP experiment as difference (in s) in the time spent in the drug‐paired compartment during post‐ and pre‐conditioning test; (2) for the locomotor activity assessment: as a distance (in cm) traveled by an animal during 15 min post‐conditioning test; (3) for the neurotransmitters' brain level: as amount of each neurotransmitter in nmol/g of tissue; (4) in hippocampus and NAc as OTRs and AVPRs mRNA expression. In all experiments, a statistically significant limit of confidence was set at *p* < 0.05.

## 3 Results

### 3.1 Impact of Mephedrone Conditioning on the Expression of Classic‐ and Social‐CPP

**FIGURE 2:**
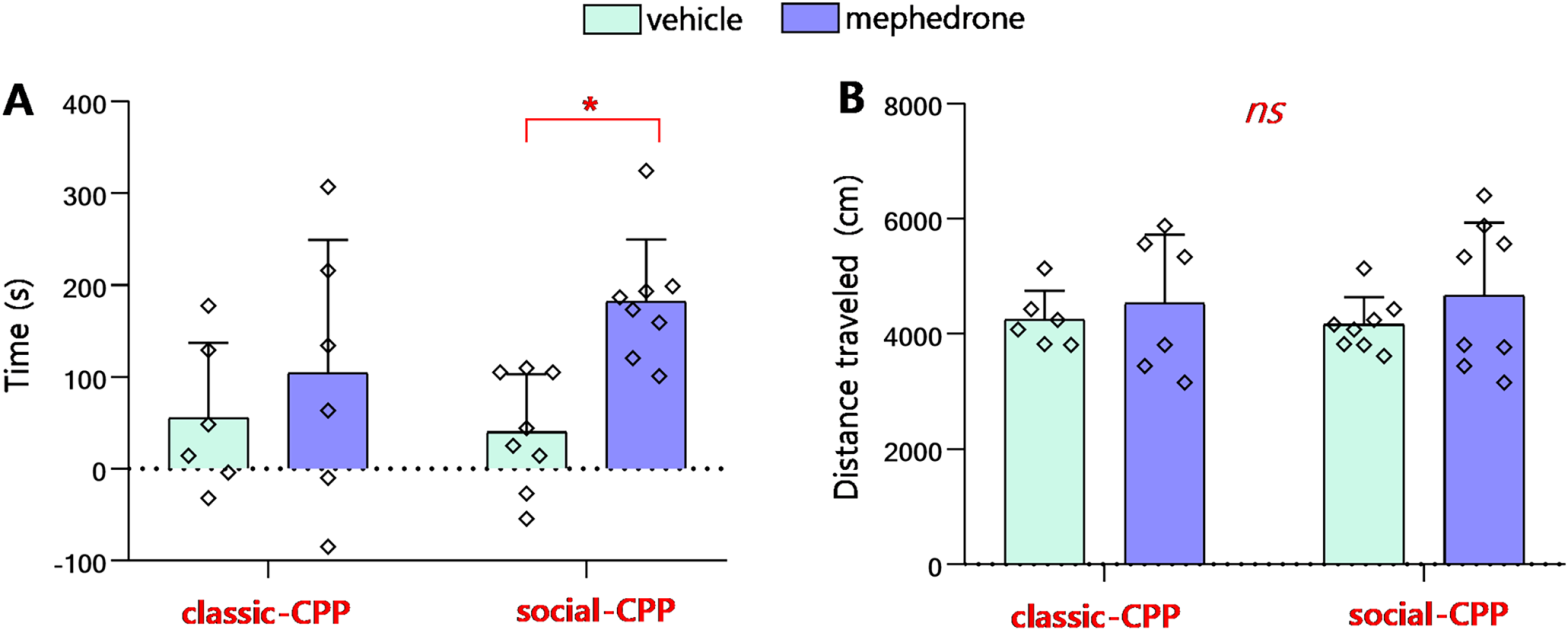
The impact of mephedrone (5 mg/kg) conditioning on (A) the expression of classic‐ and social‐CPP and (B) on locomotor activity measured during the post‐conditioning test. Data are presented as means ± SD and are expressed as (A) the difference (in s) in time spent in the drug‐paired compartment (post‐conditioning test—pre‐conditioning test) and (B) distance traveled (cm) measured during a 15‐min post‐conditioning test; *n* = 6–8 rats per group; *p < 0.05, Tukey's test.

Figure 2A shows the impact of mephedrone conditioning on the expression of mephedrone classic‐ or social‐CPP. Two‐way ANOVA with multiple comparisons showed a significant effect of mephedrone conditioning [vehicle/mephedrone conditioning: F (1, 24) = 7.568, *p* = 0.0111; classic/social conditioning: F (1, 24) = 0.8110, *p* = 0.3768; interaction: F (1, 24) = 1.807, *p* = 0.1914]. Post hoc Tukey's test showed that mephedrone treatment led to the expression of social‐CPP (versus vehicle socially‐conditioned animals) (*p* = 0.0221). Mephedrone conditioning remained ineffective in the classic version of the test. Figure 2B shows the impact of mephedrone conditioning on animals' locomotor activity measured during the post‐conditioning test and two‐way ANOVA with multiple comparisons did not show any significant effects [vehicle/mephedrone conditioning: *F* (1, 24) = 1.220, *p* = 0.2803; classic/social conditioning: *F* (1, 24) = 0.004653, *p* = 0.9462; interaction: *F* (1, 24) = 0.1035, *p* = 0.7504].

### 3.2 Impact of Classic‐ or Social‐ Mephedrone Conditioning on PFC and Hippocampal Neurotransmitters' Levels

Table 3 shows the results of two‐way ANOVA with multiple comparisons of the impact of classic‐ or social‐ mephedrone conditioning on the level of DA, 5‐HT, and NA in the PFC (Table 3A) and in the hippocampus (Table 3B).

**TABLE 3:** Results of two‐way ANOVA with multiple comparisons of the impact of classic‐ or social‐ mephedrone conditioning on the level of DA, 5‐HT, and NA in the PFC (A) and in the hippocampus (B).

Two‐way ANOVA with multiple comparisons			Significance
(A) PFC			
DA	interaction	*F* (1, 23) = 2.36, *p* = 0.0001	***
	classic/social‐CPP	*F* (1, 23) = 33.36, *p* < 0.0001	****
	vehicle/mephedrone conditioning	*F* (1, 23) = 13.35, *p* = 0.0013	**
5‐HT	interaction	*F* (1, 23) = 0.004547, *p* = 0.9468	ns
	classic/social‐CPP	*F* (1, 23) = 0.8565, *p* = 0.3643	ns
	vehicle/mephedrone conditioning	*F* (1, 23) = 1.882, *p* = 0.1834	ns
NA	interaction	*F* (1, 23) = 0.5443; *p* = 0.4681	ns
	classic/social‐CPP	*F* (1, 23) = 8.768, *p* = 0.0070	**
	vehicle/mephedrone conditioning	*F* (1, 23) = 0.3438, *p* = 0.5633	ns
(B) Hippocampus			
DA	Interaction	*F* (1, 24) = 0.1568, *p* = 0.6956	ns
	Classic/social‐CPP	*F* (1, 24) = 3.010, *p* = 0.0956	ns
	Vehicle/mephedrone conditioning	*F* (1, 24) = 0.9954, *p* = 0.3284	ns
5‐HT	Interaction	*F* (1, 24) = 1.215, *p* = 0.2812	ns
	Classic/social‐CPP	*F* (1, 24) = 10.67, *p* = 0.0033	**
	Vehicle/mephedrone conditioning	*F* (1, 24) = 0.1006, *p* = 0.7539	ns
NA	Interaction	*F* (1, 24) = 4.389, *p* = 0.0469	*
	Classic/social‐CPP	*F* (1, 24) = 66.16, *p* < 0.0001	**
	Vehicle/mephedrone conditioning	*F* (1, 24) = 0.05711, *p* = 0.8132	ns

* *p* < 0.05.

** *p* < 0.01.

*** *p* < 0.001.

**** *p* < 0.0001.

**FIGURE 3:**
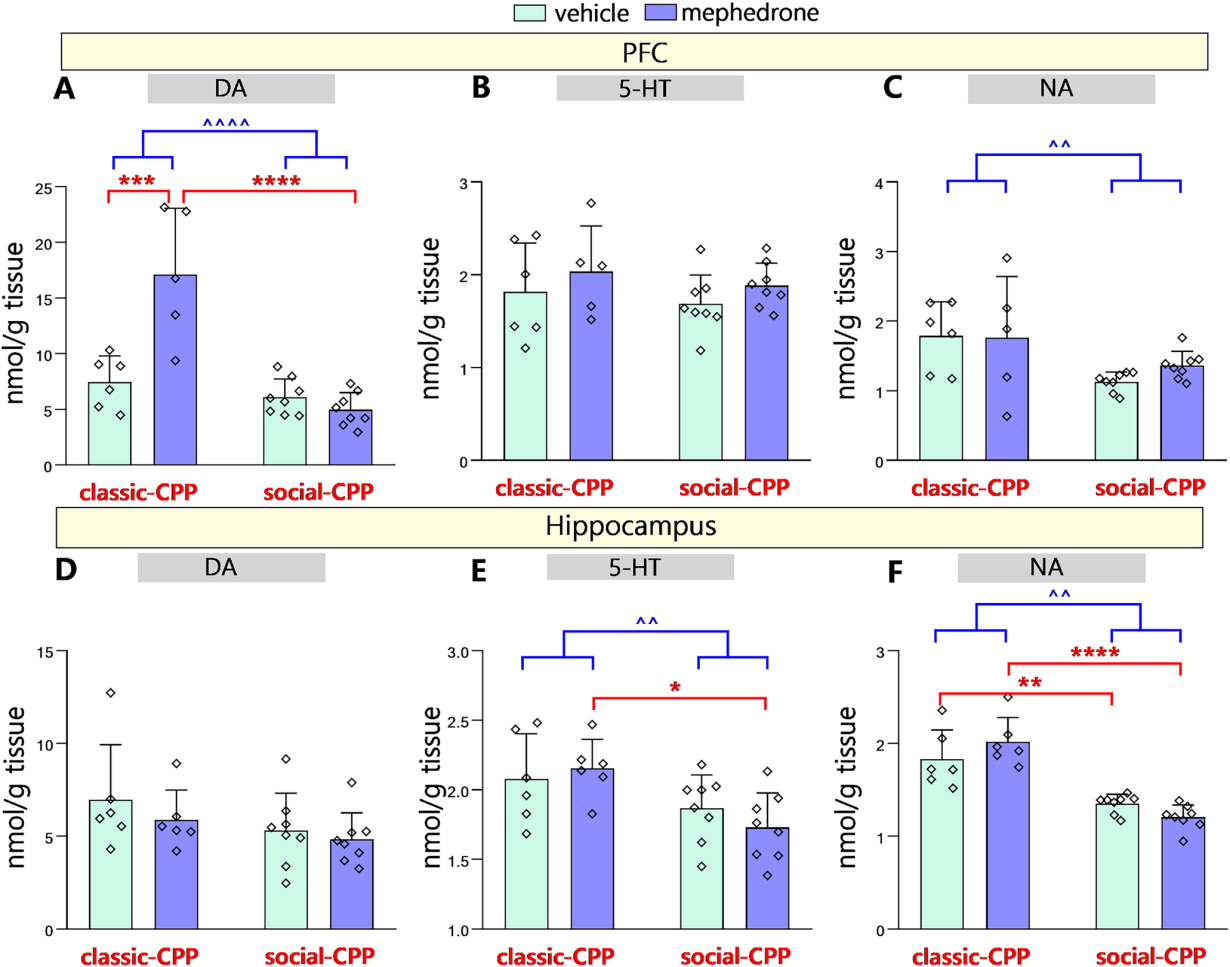
Impact of classic‐ or social‐ mephedrone conditioning on the level of DA, 5‐HT, and NA in the PFC (A–C) and in the hippocampus (D–F). Data are presented as means ± SD and are expressed as the amount of each neurotransmitter in nmol/g of tissue; *n* = 5–8; ^^ *p* < 0.01, ^^^^ *p* < 0.0001 (classic/social‐CPP, main ANOVA effect); *p < 0.05, **p < 0.01, ***p < 0.001, and ****p < 0.0001 (Tukey's test).

In the PFC, the results showed a significant effect of the type of conditioning (social/classic), of the treatment (vehicle/mephedrone conditioning) and interaction between the type of conditioning and treatment for DA. There was also a significant effect of the type of conditioning (social/classic) for NA (Table 3A). Post hoc Tukey's test showed that classic mephedrone conditioning led to an increase of PFC DA level (versus vehicle classic‐CPP) (*p* = 0.001) and this effect was reversed by the incorporation of social context to mephedrone conditioning in the social‐CPP paradigm (*p* < 0.0001) (Figure 3A). Post hoc Tukey's test did not show any significant changes in 5‐HT and NA levels (Figure 3B,C).

In the hippocampus, there was a significant effect of the type of the conditioning (social/classic) for 5‐HT and a significant effect of the type of the conditioning (social/classic), as well as of the interaction between the type of the conditioning and treatment for NA (Table 3B). Post hoc Tukey's test did not show any significant changes in DA levels (Figure 3D); however, it showed a significant decrease in 5‐HT hippocampal level in mephedrone socially‐conditioned animals (versus mephedrone classically‐conditioned animals) (*p* = 0.0242) (Figure 3E). In addition, there was a significant effect of the type of the conditioning (regardless of the conditioning treatment) on the level of NA which was decreased in socially‐conditioned animals with vehicle (versus classically‐conditioned animals with vehicle, *p* = 0.0014), as well as with mephedrone (versus classically‐conditioned animals with mephedrone, *p* < 0.0001) (Figure 3F).

### 3.3 Impact of Classic‐ or Social‐Conditioning and Mephedrone Treatment on OT and AVP Plasma Concentration

**FIGURE 4:**
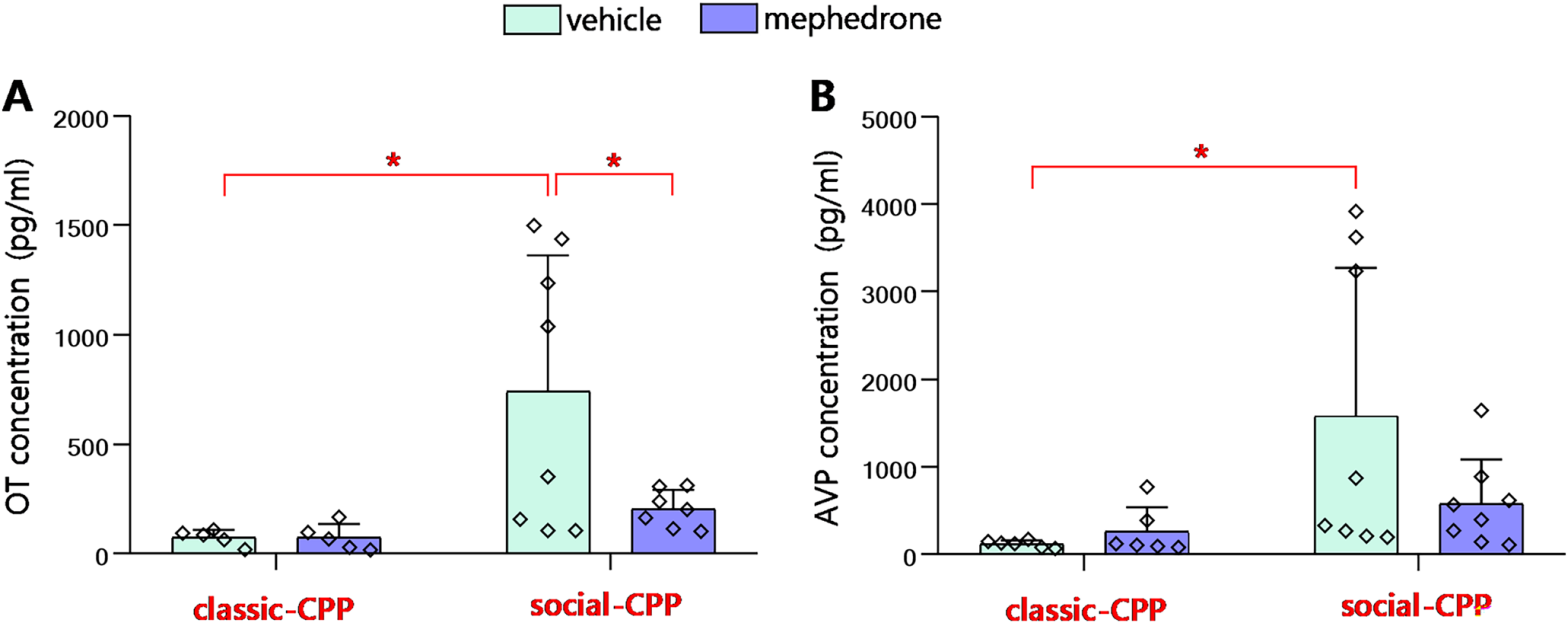
The impact of subchronic mephedrone treatment (5 mg/kg, once a day, for 6 days) in classic‐ or social‐CPP protocol on OT and AVP plasma concentration (blood samples collection was performed 24 h after last mephedrone/vehicle treatment). Vehicle socially versus classically conditioned animals (*p < 0.05); mephedrone socially conditioned versus vehicle socially conditioned animals (*p < 0.05); post hoc Tukey's test; *n* = 5–8 (for OT) and 6–8 (for AVP).

Figure 4 shows the effects of mephedrone classic‐ and social‐conditioning on OT (Figure 4A) and AVP (Figure 4B) plasma levels. Two‐way ANOVA for OT [vehicle/mephedrone conditioning: F (1, 21) = 3.255, *p* = 0.0856; classic/social conditioning: F (1, 21) = 7.261, *p* = 0.0136; interaction: F (1, 21) = 3.281, *p* = 0.0844] showed a significant effect of the type of conditioning. Post hoc multiple comparisons Tukey's test showed a significant increase in OT plasma level in vehicle socially‐conditioned animals versus vehicle classically‐conditioned animals (*p* = 0.0194) and a significant decrease in OT plasma level in mephedrone socially‐conditioned animals versus vehicle‐socially conditioned animals (*p* = 0.0435). Two‐way ANOVA for AVP [vehicle/mephedrone conditioning: F (1, 24) = 1.378, *p* = 0.2519; classic/social conditioning: F (1, 24) = 5.912, *p* = 0.0229; interaction: F (1, 24) = 2.430, *p* = 0.1322] also showed a significant effect of the type of conditioning. Post hoc multiple comparisons Tukey's test showed a significant increase in OT plasma level in vehicle socially‐conditioned animals versus vehicle classically‐conditioned animals (*p* = 0.0437).

### 3.4 Impact of Subchronic Mephedrone Treatment on OTR and AVPV_1A_R Gene Expression in the Hippocampus and in the NAc

**FIGURE 5:**
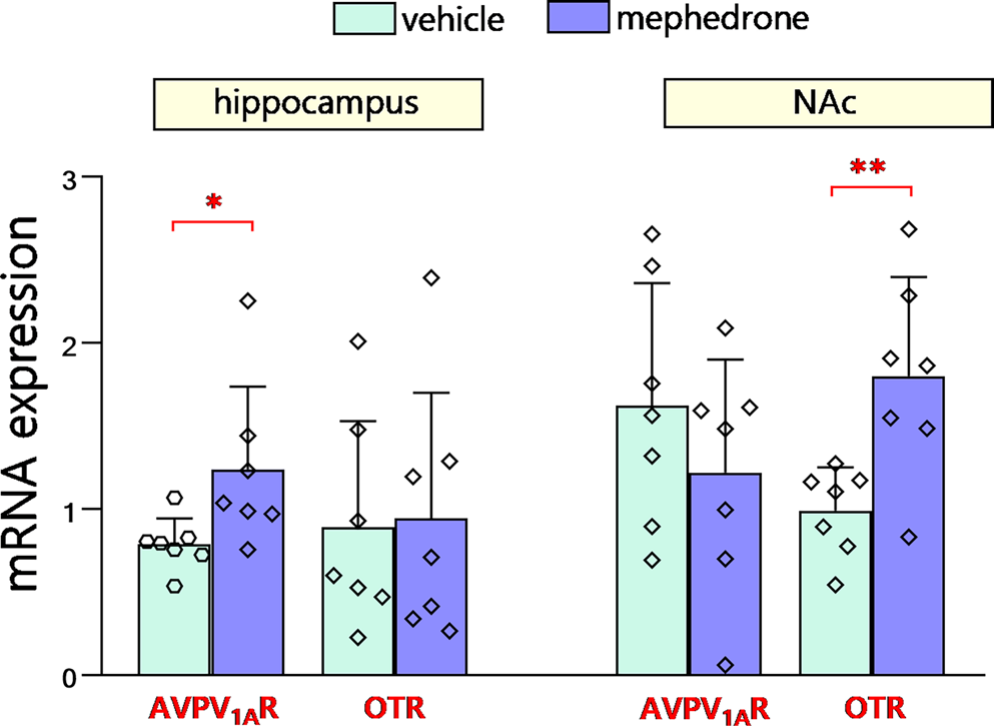
The impact of subchronic mephedrone treatment (5 mg/kg, once a day, for 6 days) on the OTRs and AVPRs gene expression in the hippocampus and in the NAc (decapitation and dissection was performed 24 h after last mephedrone/vehicle treatment). Mephedrone (5 mg/kg) versus vehicle treated animals, *p < 0.05; **p < 0.01.

The statistical results of the subchronic mephedrone treatment on OTRs and AVPV_1A_Rs gene expression in the hippocampus and in the NAc have been presented in Table 4. The results showed that mephedrone treatment (5 mg/kg, once a day, for 6 days) significantly increased the AVPV_1A_R gene expression in the hippocampus (*p* = 0.0403) and increased the OTR gene expression in the NAc (*p* = 0.0064) (Figure 5).

**TABLE 4:** The results of the statistical analysis of the impact of subchronic mephedrone treatment (5 mg/kg, once a day, for 6 days) on the OTRs and AVPRs gene expression in the hippocampus and in the NAc (decapitation and dissection was performed 24 h after last mephedrone/vehicle treatment).

Brain region	Receptor	*t*‐test (unpaired, two‐tailed)	Significance
Hippocampus	AVPV_1A_R	***p* = 0**.**0403**; *t* = 2.299, df = 12	*
	OTR	*p* = 0.8925; *t* = 0.1381, df = 12	ns
NAc	AVPV_1A_R	*p* = 0.3111; *t* = 1.057, df = 12	ns
	OTR	***p* = 0**.**0064**; *t* = 3.293, df = 12	**

*Note:* The *t*‐test (unpaired, two‐tailed) has been performed between mephedrone (5 mg/kg) versus vehicle treated animals.

* *p* < 0.05 (in bold).

** *p* < 0.01 (in bold).

### 3.5 Impact of Acute Treatment With OTR Ligands (Agonist: Carbetocin and Antagonist: L‐368,899), AVP and AVPR Ligand (Antagonist: SR49059) on the Expression of Mephedrone‐Induced Social CPP

**FIGURE 6:**
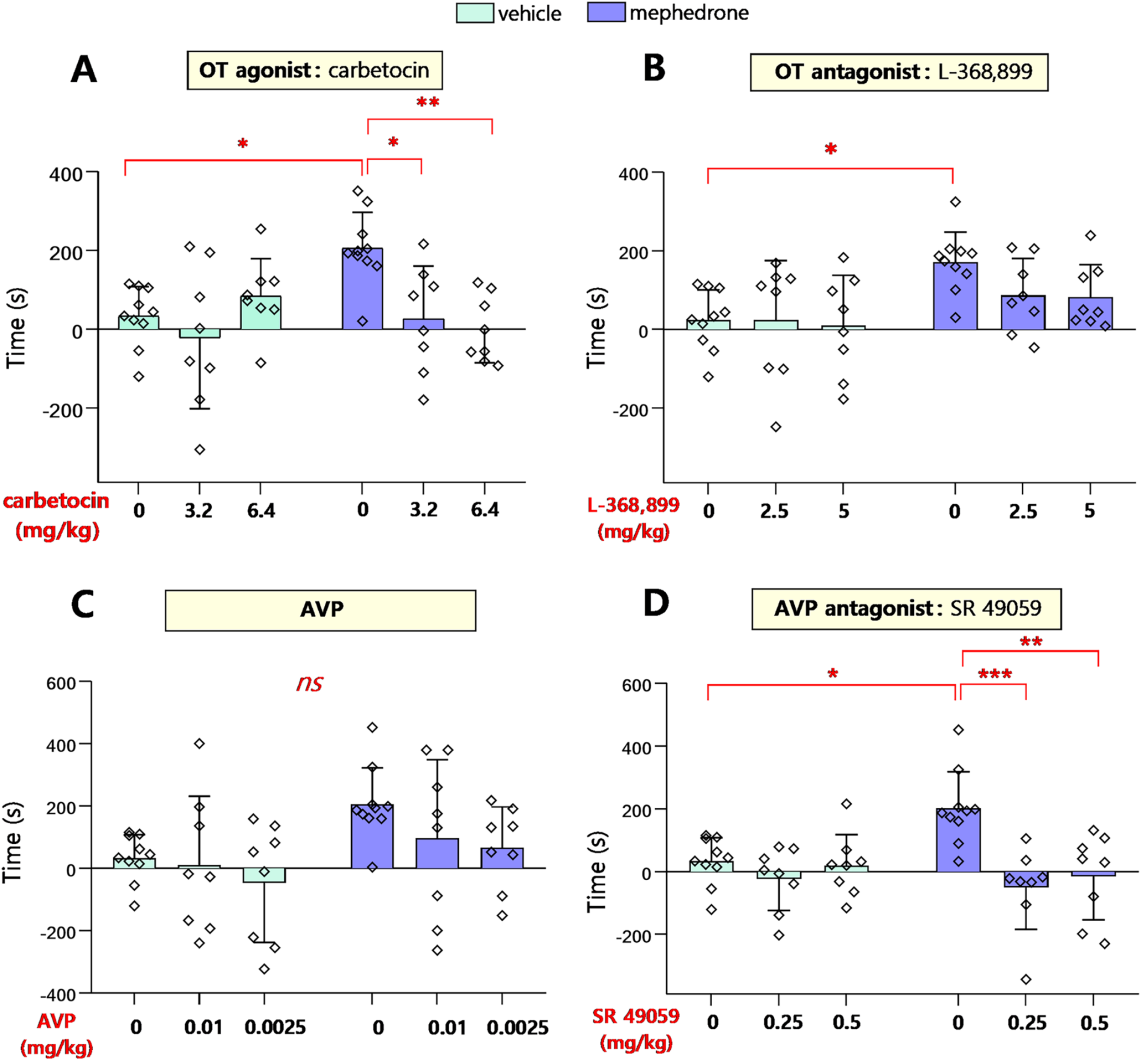
The impact of (A) carbetocin; (B) L‐368,899; (C) AVP and (D) SR49059 on the expression of mephedrone‐induced social‐conditioned place preference (social‐CPP). Data are presented as means ± SD and are expressed as the difference (in s) in time spent in the drug‐paired compartment (post‐conditioning test—pre‐conditioning test); *n* = 8–10 rats per group; * *p* < 0.05, **p < 0.01; ***p < 0.001 (Tukey's test).

Table 5 shows the results of two‐way ANOVA with multiple comparisons of the impact of acute post‐conditioning treatment with carbetocin, L‐368,899, AVP and SR49059 on the expression of mephedrone‐induced social CPP. Firstly, two‐way ANOVA analysis revealed a significant effect of post‐conditioning treatment with carbetocin and the interaction between conditioning and post‐conditioning treatment. Post hoc Tukey's test showed that mephedrone was able to induce social‐CPP (in comparison to vehicle‐conditioned and vehicle‐post‐conditioning treated animals, *p* = 0.0165) and carbetocin successfully reversed this effect in both of the tested doses (3.2 and 6.4 mg/kg) (in comparison to mephedrone‐conditioned and vehicle‐post‐conditioning treated animals, *p* = 0.0197 and *p* = 0.0047, respectively) (Figure 6A). Secondly, two‐way ANOVA showed a significant effect for the conditioning treatment but no effect for the post‐conditioning treatment with L‐368,899. Post hoc Tukey's test confirmed that mephedrone induced social‐CPP (in comparison to vehicle‐conditioned and vehicle‐post‐conditioning treated animals, *p* = 0.0284); however, post‐conditioning treatment with L‐368,899 did not influence this effect (Figure 6B). Thirdly, two‐way ANOVA showed a significant effect of conditioning treatment but post hoc Tukey's test did not reveal any effect of the post‐conditioning treatment with AVP (Figure 6C). Finally, two‐way ANOVA revealed a significant effect of post‐conditioning treatment with SR49059 and the interaction between conditioning and post‐conditioning treatment. Post hoc Tukey's test showed the ability of mephedrone to induce social‐CPP (in comparison to vehicle‐conditioned and vehicle‐post‐conditioning treated animals, *p* = 0.0171) and SR49059 was able to reverse this effect in both of the tested doses (0.25 and 0.5 mg/kg) (in comparison to mephedrone‐conditioned and vehicle‐post‐conditioning treated animals, *p* = 0.0002 and *p* = 0.002, respectively) (Figure 6D).

**TABLE 5:** Results of two‐way ANOVA with multiple comparisons of the impact of acute post‐conditioning treatment with carbetocin, L‐368,899, AVP and SR49059 on the expression of mephedrone‐induced social CPP.

Social‐CPP			
Post conditioning treatment	Two‐way ANOVA		Significance
Carbetocin	Interaction	*F* (2, 46) = 5.761, *p* = 0.0058	**
	Post‐conditioning treatment	*F* (2, 46) = 5.045, *p* = 0.0104	*
	Vehicle/mephedrone conditioning	*F* (1, 46) = 2.040, *p* = 0.1600	ns
L‐368,899	Interaction	*F* (2, 46) = 0.9255, *p* = 0.4036	ns
	Post‐conditioning treatment	*F* (2, 46) = 1.308, *p* = 0.2802	ns
	Vehicle/mephedrone conditioning	*F* (1, 46) = 10.77, *p* = 0.0020	**
AVP	Interaction	*F* (2, 46) = 0.3079, *p* = 0.7365	ns
	Post‐conditioning treatment	*F* (2, 46) = 1.918, *p* = 0.1584	ns
	Vehicle/mephedrone conditioning	*F* (1, 46) = 6.849, *p* = 0.0120	*
SR49059	Interaction	*F* (2, 46) = 4.950, *p* = 0.0113	*
	Post‐conditioning treatment	*F* (2, 46) = 9.628, *p* = 0.0003	***
	Vehicle/mephedrone conditioning	*F* (1, 46) = 1.317, *p* = 0.2571	ns

* *p* < 0.05.

** *p* < 0.01.

*** *p* < 0.001.

### 3.6 Impact of Acute Treatment With OTRs Ligands (Carbetocin and L‐368,899), AVP and AVPRs Ligand (SR49059) on Animals' Locomotor Activity

**FIGURE 7:**
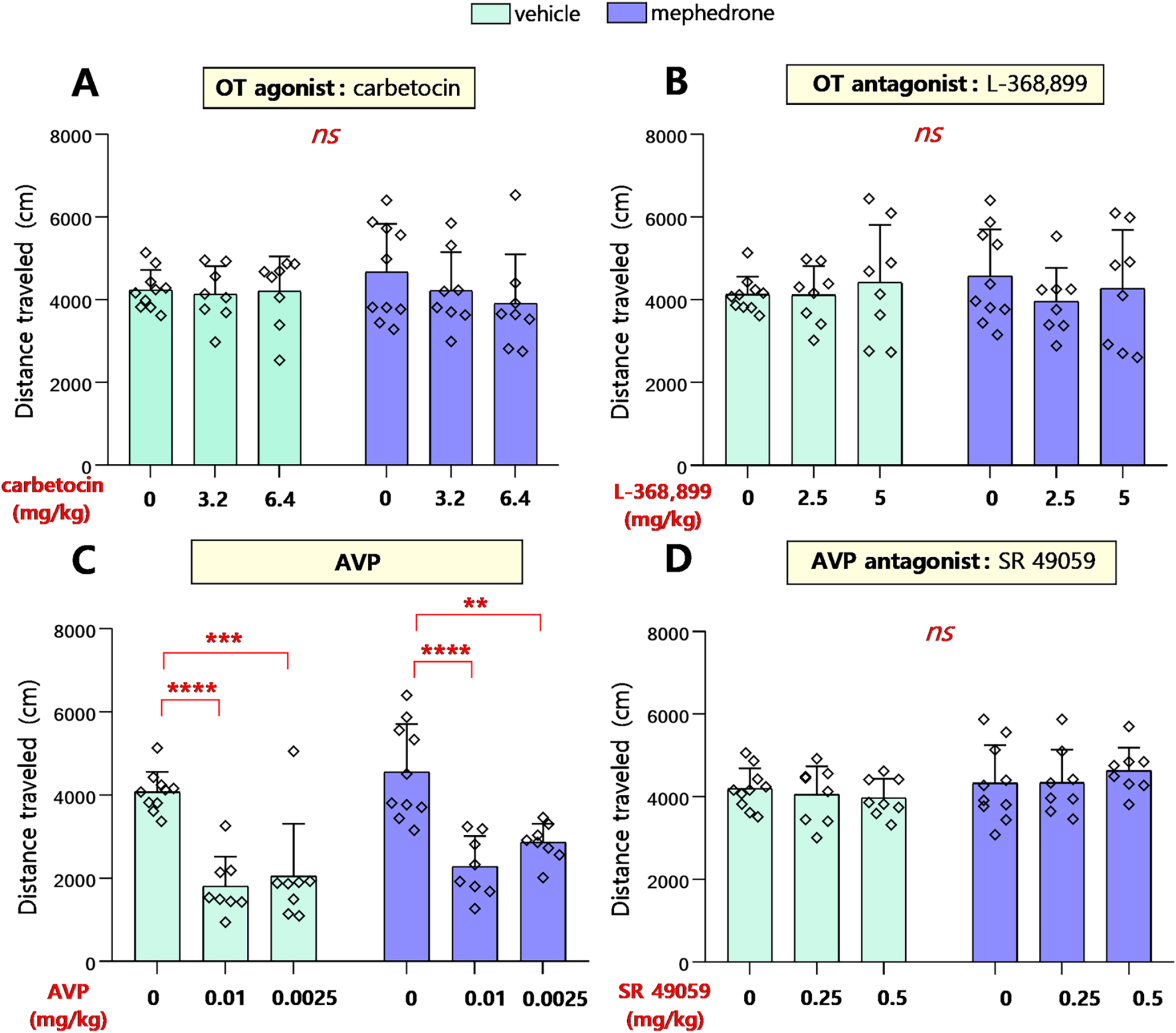
The impact of (A) carbetocin; (B) L‐368,899; (C) AVP and (D) SR49059 on animals' mobility. Data are presented as means ± SD of distance traveled (cm) measured during a 15‐min post‐conditioning test—24 h after last mephedrone injection and (A) 15 min after carbetocin injection; (B) 45 min after L‐368,899 injection; (C) 20 min after AVP injection; (D) 30 min after SR 49059 injection; **p < 0.01; ***p < 0.001; ****p < 0.0001 (Tukey's test).

Table 6 shows the results of two‐way ANOVA with multiple comparisons of the impact of acute treatment with OTRs ligands, AVP and AVPRs ligands on animals' mobility measured during the post‐conditioning test. The analysis revealed a significant effect of acute post‐conditioning treatment with AVP on animals' locomotion for both saline‐ and mephedrone‐conditioned animals. Other tested ligands (carbetocin, L‐368,899 and SR49059) remained without effect on animals' mobility, which was also shown as no significant changes in post hoc Tukey's test (Figure 7A,B,D). However, post hoc Tukey's test showed that acute post‐conditioning treatment with both of the tested doses of AVP (0.01 and 0.0025 mg/kg) led to a significant decrease in animals' mobility in both saline‐ (*p* < 0.0001, *p* = 0.0001) and mephedrone‐conditioned animals (*p* < 0.0001, *p* = 0.0018, respectively) (Figure 7C).

**TABLE 6:** Results of two‐way ANOVA with multiple comparisons of the impact of carbetocin, L‐368,899, AVP and SR49059 on animals' mobility measured during a 15‐min post‐conditioning test—24 h after last mephedrone injection and 15 min after carbetocin injection; 45 min after L‐368,899 injection; 20 min after AVP injection; 30 min after SR 49059 injection.

Locomotor activity			
Post conditioning treatment	Two‐way ANOVA		Significance
Carbetocin	Interaction	*F* (2, 46) = 0.7036, *p* = 0.5000	ns
	Post‐conditioning treatment	*F* (2, 46) = 0.9156, *p* = 0.4074	ns
	Vehicle/mephedrone conditioning	*F* (1, 46) = 0.07716, *p* = 0.7824	ns
L‐368,899	Interaction	*F* (2, 46) = 0.5119, *p* = 0.6027	ns
	Post‐conditioning treatment	*F* (2, 46) = 0.5056, *p* = 0.6065	ns
	Vehicle/mephedrone conditioning	*F* (1, 46) = 0.02719, *p* = 0.8698	ns
AVP	Interaction	*F* (2, 46) = 0.2121, *p* = 0.8097	ns
	Post‐conditioning treatment	*F* (2, 46) = 36.63, *p* < 0.0001	****
	Vehicle/mephedrone conditioning	*F* (1, 46) = 6.068, *p* = 0.0176	*
SR49059	Interaction	*F* (2, 46) = 0.6729, *p* = 0.5152	ns
	Post‐conditioning treatment	*F* (2, 46) = 0.09600, *p* = 0.9086	ns
	Vehicle/mephedrone conditioning	*F* (1, 46) = 3.601, *p* = 0.0640	ns

* *p* < 0.05.

**** *p* < 0.0001.

## 4 Discussion

The present study builds upon our previous findings demonstrating that conditioning with a low dose of mephedrone (5 mg/kg), which failed to induce a preference in the classic‐CPP paradigm, effectively produced a rewarding effect in the social‐CPP version of the test [10]. These results were successfully replicated also in this study, underscoring the critical role of social context in modulating the behavioral effects of mephedrone. Consequently, a carefully designed set of experiments was conducted to investigate the molecular mechanisms underlying this context‐dependent response.

Numerous studies have provided evidence that mephedrone treatment influences DA, 5‐HT, and NA levels across various brain regions. For instance, mephedrone self‐administration produced sex‐dependent neurochemical adaptations, characterized by reductions in hippocampal and prefrontal 5‐HT in both sexes, selective decreases in hippocampal NA in males, and differential DA/NA modulation in females [45, 46]. Furthermore, pretreatment during adolescence increased DA and 5‐HT levels in the frontal cortex of adult rats [47], while binge [48] and chronic [17] treatment showed no significant effects on hippocampal 5‐HT or DA levels. In contrast, acute administration elevated frontal cortical DA [49], whereas acute repeated dosing decreased hippocampal 5‐HT [50].

Regrettably, none of the above‐mentioned studies have addressed whether the observed neurochemical changes are influenced by the social context. At the same time, the involvement of OT and AVP in both social‐context‐dependent and ‐independent drug reward is well established [18]; however, their role in mephedrone‐induced reward remains unclear. Therefore, the present study aimed to address this gap by evaluating the effects of classic versus social mephedrone conditioning on neurotransmitter levels in the PFC and hippocampus, as well as on plasma levels of OT and AVP.

The present findings indicate that the type of conditioning exerts a significant influence on the observed neurochemical changes. In the classic CPP paradigm, mephedrone induced a robust increase in PFC DA levels; however, this neurochemical effect did not translate into behavioral reinforcement, as mephedrone failed to induce CPP in the classic design. Notably, the elevation of PFC DA was completely abolished under social mephedrone‐conditioning, suggesting that the presence of social cues dampens mephedrone's ability to recruit prefrontal dopaminergic signaling. Conversely, the social context in combination with mephedrone was associated with marked reductions in hippocampal 5‐HT and NA (in comparison to mephedrone classically‐conditioned animals). This suggests that while solitary drug‐context associations primarily engage dopaminergic circuits linked to reward, social conditioning shifts the neurochemical profile toward serotonergic and noradrenergic adaptations in the hippocampus, thereby reshaping the neural basis of mephedrone reinforcement. Interestingly, analysis of the main conditioning effects revealed that the incorporation of a social element into the paradigm itself resulted in decreased PFC DA and NA levels, as well as reduced hippocampal 5‐HT and NA levels, irrespective of mephedrone or vehicle treatment. This pattern suggests that the presence of social cues may dampen baseline monoaminergic activity in key cortical and hippocampal regions, potentially reflecting a regulatory or adaptive effect of social context on neurochemical signaling.

As these results strongly suggest that the observed neurochemical effects are modulated by the social context, it became essential to examine whether similar context‐dependent alterations would also be reflected in OT and AVP levels. The rationale for measuring plasma OT and AVP levels in the present study was to assess whether the social component of the CPP paradigm and/or repeated mephedrone exposure were associated with systemic neuroendocrine changes. Although plasma OT and AVP concentrations cannot be considered direct markers of central peptide release or direct mechanistic correlates of CPP effects, previous studies on MDMA, including randomized controlled trials [51] and studies conducted in club‐going populations [52], have demonstrated drug‐induced changes in peripheral OT and AVP levels related to prosocial and neuroendocrine effects. In our paradigm, social conditioning increased plasma OT and AVP in vehicle‐treated animals, whereas mephedrone selectively prevented the social‐context‐related increase in plasma OT. Accordingly, our plasma OT/AVP data should be interpreted as complementary evidence that the social component of the paradigm engages OT/AVP‐related neuroendocrine responses, which are differentially modulated by mephedrone, without indicating that these peripheral changes directly mediate CPP expression.

Therefore, to better understand the involvement of these neuropeptides in mephedrone‐reward, the evaluation of the OTRs and AVPV_1A_Rs gene expression was performed in the hippocampus and in the NAc [53, 54]. Both the hippocampus and the NAc are involved in OT‐ and AVP‐mediated regulation of reward‐ and context‐related behavioral responses. Specifically, the NAc has been identified as a key component of the reward circuitry in which OTRs activation modulates social reward and social approach, linking this structure to both reward‐ and social‐related behaviors [55, 56]. Moreover, hippocampal OTRs and AVPRs have been implicated in social recognition and memory processes, supporting the relevance of this structure for context‐dependent behavioral responses, including those associated with addiction [57, 58, 59]. Our analysis showed that subchronic mephedrone treatment led to an increased expression of AVPV_1A_Rs in the hippocampus and of OTRs in the NAc.

This surprising counterintuitive effect clearly indicates a divergence between the peripheral and central regulation of the OT and AVP systems. While plasma levels of OT and AVP reflect acute, socially dependent changes, the altered receptor expression in the hippocampus and NAc suggests that mephedrone may primarily engage central adaptive mechanisms [60]. A similar effect was observed in methamphetamine self‐administration, which led to an increased plasma OT yet reduced OTR‐immunoreactive fiber density in the NAc core, directly illustrating a dissociation between peripheral peptide levels and central receptor adaptations [61]. Furthermore, in our study, the observed upregulation of AVPV_1A_Rs in the hippocampus may be linked to drug‐induced modulation of contextual memory and emotional processing [62, 63], whereas the increased OTR expression in the NAc points to a role in reward‐related plasticity [64]. Taken together, these findings highlight that mephedrone not only modulates circulating neuropeptides but also reshapes receptor‐mediated signaling within specific brain regions, providing a potential mechanism through which social and pharmacological factors converge to influence reward processing and behavioral outcomes.

Therefore, due to the above‐mentioned inconclusive OT/AVP effect direction, it was essential to determine how OT/AVP transmission is reflected in behavioral effects of mephedrone in rats. Considering (1) the ambiguous biochemical (plasma OT/AVP levels) and molecular (receptors gene expression) results and (2) strong evidence that drug reward may be attenuated by both activation or inhibition of OT/AVP transmission [18], it was vital to test both OTRs/AVPRs agonists as well as antagonists in the social‐CPP. The results showed that mephedrone‐induced social‐CPP was successfully reversed by acute treatment with OTRs agonist carbetocin and with AVPV_1A_Rs antagonist SR49059. OTRs antagonist L‐368,899 and AVP remained ineffective; however, AVP also led to a significant decrease in animals' locomotion.

These pharmacological data align with previous evidence showing that activation of OT transmission can counteract the rewarding effects of various drugs of abuse [18]. Specifically, the ability of carbetocin to abolish mephedrone‐induced social‐CPP supports the hypothesis that OTR stimulation represents a protective mechanism limiting drug reward, particularly in socially modulated contexts [30, 35]. Interestingly, the reversal of social‐CPP by the AVPV_1A_R antagonist SR49059 indicates that vasopressin signaling through V_1A_ receptors may facilitate mephedrone reward. This observation is in agreement with earlier reports that AVPV_1A_R blockade interferes with OT‐mediated attenuation of stimulant reward in rodents [41], highlighting a complex interplay between these two neuropeptidergic systems. It is also consistent with previous studies on MDMA, which demonstrated that SR49059 was able to block MDMA‐induced prosocial effects, such as adjacent lying in rats [27].

Importantly, because carbetocin and SR49059 were administered peripherally, their behavioral effects should not be interpreted as direct evidence of OTR activation within the NAc or AVPV_1A_R blockade within the hippocampus, respectively. Although peripherally administered OT/AVP‐related ligands can influence reward‐related behaviors (see [18]), these effects may involve limited central access and/or indirect peripheral‐to‐central mechanisms [65, 66]. Therefore, the increased OTR mRNA expression in the NAc and AVPV_1A_R mRNA expression in the hippocampus should be interpreted as transcriptional adaptations within reward‐ and memory‐related regions, rather than as evidence that the ligands acted specifically at these sites. Accordingly, the pharmacological and gene expression findings should be viewed as complementary but mechanistically distinct evidence for the involvement of oxytocinergic and vasopressinergic signaling in mephedrone‐induced social reward.

The lack of effect of the OTR antagonist L‐368,899 suggests that basal OTR signaling is not sufficient to drive mephedrone reward in the social‐CPP paradigm, but rather that the critical factor is the potentiation of OTR activity, which is capable of shifting the balance towards reduced reward sensitivity. Similarly, the absence of significant behavioral effects following AVP administration points to a limited role of peripheral AVP elevation per se in mediating the rewarding properties of mephedrone. The observed reduction in locomotor activity after AVP treatment may instead reflect nonspecific sedative or motivational effects unrelated to drug‐context conditioning.

Taken together, these findings emphasize that mephedrone‐induced social reward depends on a finely tuned balance between OT and AVP signaling, where OTR activation exerts an inhibitory influence, while AVPV_1A_R activity appears to promote mephedrone reinforcement. Importantly, these effects cannot be explained solely by changes in peripheral peptide concentrations, but rather reflect central adaptations at the receptor level within key reward‐ and memory‐related brain regions such as the NAc and hippocampus. This dual mechanism—peripheral neuroendocrine responses combined with central receptor plasticity—may underlie the strong modulatory impact of the social environment on mephedrone reward.

Although our study provides novel insights into the role of OT and AVP signaling in mephedrone‐induced social reward, a clear limitation is that we did not investigate the potential contribution of AVPV_1B_Rs to this process. Previous findings indicate that pharmacological blockade of these receptors with SSR149415 produces effects opposite to those observed following AVPV_1A_R inhibition, including attenuation of ethanol intake, prevention of morphine‐CPP acquisition, and suppression of heroin‐ and nicotine‐induced reinstatement [67, 68, 69, 70, 71, 72]. These data strongly suggest that AVPV_1B_R signaling may exert a modulatory influence on drug reward processes, potentially acting in a protective manner against reinforcement. Therefore, the absence of experiments involving AVPV_1B_R ligands in our study limits the scope of interpretation, as it remains unclear whether the balance between AVPV_1A_R‐ and AVPV_1B_R‐mediated signaling contributes to the observed context‐dependent effects of mephedrone. Therefore, an important next step will be to evaluate the role of AVPV_1B_Rs in the social‐CPP paradigm. Another possible limitation of the present study is that it focuses on OTR and AVPV_1A_R gene expression without assessing protein levels or functional receptor activity, which limits conclusions about actual OT/AVP system engagement. Additionally, the lack of investigation into sex‐dependent effects, given the modulation of these systems by sex hormones [73], restricts the generalizability of the findings.

## 5 Conclusions

In summary, the present study demonstrates that mephedrone‐induced social reward is critically modulated by the social context, as the type of conditioning differently affected central neurotransmitter levels. Furthermore, it may be concluded that the addictive potential of mephedrone may be strongly enhanced by social context, which is particularly relevant given the recreational patterns of its use. In addition, mephedrone‐induced social reward appears to involve a finely tuned interplay between OT and AVP signaling. Specifically, OTR activation limits drug reward, while AVPV_1A_R activity promotes it, highlighting a dual mechanism that integrates peripheral neuroendocrine responses with central receptor adaptations in key reward‐ and memory‐related brain regions. Thus, the present findings suggest that the addictive properties of mephedrone cannot be fully understood based solely on its direct psychostimulant action, but should also be considered in relation to social‐context‐dependent neurobiological mechanisms. These findings underscore the importance of considering both social environment and neuropeptidergic systems in understanding psychostimulant reinforcement. Importantly, the results point to potential therapeutic targets—particularly OTR and AVPV_1A_R modulation—for mitigating social‐context‐dependent drug reward. Overall, these results support the conclusion that mephedrone possesses context‐dependent addictive properties, with social interaction acting as a critical factor that can facilitate the expression of its rewarding effects. Nevertheless, it should be highlighted that these conclusions should be interpreted with caution, as peripheral OT/AVP changes and central receptor adaptations provide complementary, but not direct, evidence for the neuropeptidergic regulation of mephedrone‐induced social reward.

## Funding

Research was funded by: (1) the National Science Centre (Poland), Grant Identification Number: NCN 2020/37/N/NZ7/01564 (to OWD)—funding of all presented experiments (except for the results shown in Figure 6A,C and Figure 7A,C) and (2) the intramural grant for young scientists of Medical University of Lublin (Poland) Pbmb 211 (to OWD)—results presented in Figure 6A,C and Figure 7A,C.

## Ethics Statement

All experiments: (1) adhered to the ARRIVE guidelines; (2) were conducted in accordance with the National Institutes of Health Guidelines for the Care and Use of Laboratory Animals, as well as the European Community Council Directive for the Care and Use of Laboratory Animals of 22 September 2010 (2010/63/EU); and (3) received approval from the Local Ethics Committee in Lublin, Poland (Permissions No: 59/2021 and 52/2022).

## Conflicts of Interest

The authors declare no conflicts of interest.

## Data Availability

The data that support the findings of this study are available from the corresponding author upon reasonable request.

## References

[1] European Union Drug Agency (EUDA) , European Drug Report 2025. Accessed August 22, 2025 (2025), https://www.euda.europa.eu/publications/eu‐drug‐markets/new‐psychoactive‐substances/distribution‐and‐supply/synthetic‐cathinones_en?utm_source=chatgpt.com.

[2] J. Mennis , G. J. Stahler , and M. J. Mason , “Risky Substance Use Environments and Addiction: A New Frontier for Environmental Justice Research,” International Journal of Environmental Research and Public Health 13, no. 6 (2016): 607, 10.3390/ijerph13060607. 27322303 PMC4924064

[3] S. S. H. Aghaii , A. Kamaly , and M. Esfahani , “Meta‐Analysis of Individual and Environmental Factors That Influence People's Addiction Tendencies,” International Journal of High Risk Behaviors and Addiction 1, no. 3 (2012): 92–99, 10.5812/ijhrba.5330. 24971243 PMC4070113

[4] M. Hosseinbor , S. M. Yassini Ardekani , S. Bakhshani , and S. Bakhshani , “Emotional and Social Loneliness in Individuals With and Without Substance Dependence Disorder,” International Journal of High Risk Behaviors and Addiction 3, no. 3 (2014): e22688, 10.5812/ijhrba.22688. 25632385 PMC4295122

[5] B. Simons‐Morton and R. S. Chen , “Over Time Relationships Between Early Adolescent and Peer Substance Use,” Addictive Behaviors 31, no. 7 (2006): 1211–1223, 10.1016/j.addbeh.2005.09.006. 16229958

[6] S. L. Cole , R. S. Hofford , D. J. Evert , P. J. Wellman , and S. Eitan , “Social Influences on Morphine Conditioned Place Preference in Adolescent Mice,” Addiction Biology 18 (2013): 274–285, 10.1111/j.1369-1600.2011.00426.x. 22339796

[7] L. Ramos , C. Hicks , A. Caminer , J. Goodwin , and I. S. McGregor , “Oxytocin and MDMA (‘Ecstasy’) Enhance Social Reward in Rats,” Psychopharmacology 232 (2015): 2631–2641, 10.1007/s00213-015-3899-9. 25772337

[8] K. J. Thiel , A. C. Okun , and J. L. Neisewander , “Social Reward‐Conditioned Place Preference: A Model Revealing an Interaction Between Cocaine and Social Context Rewards in Rats,” Drug and Alcohol Dependence 96 (2008): 202–212, 10.1016/j.drugalcdep.2008.02.013. 18430522 PMC2488154

[9] K. J. Thiel , F. Sanabria , and J. L. Neisewander , “Synergistic Interaction Between Nicotine and Social Rewards in Adolescent Male Rats,” Psychopharmacology 204 (2009): 391–402, 10.1007/s00213-009-1470-2. 19224200 PMC2831774

[10] O. Wronikowska , M. Zykubek , Ł. Kurach , A. Michalak , A. Boguszewska‐Czubara , and B. Budzyńska , “Vulnerability Factors for Mephedrone‐Induced Conditioned Place Preference in Rats‐The Impact of Sex Differences, Social‐Conditioning and Stress,” Psychopharmacology 238 (2021): 2947–2961, 10.1007/s00213-021-05910-y. 34268586 PMC8455394

[11] European Union Drug Agency (EUDA) and Europol , EU Drug Market: New Psychoactive Substances—Distribution and Supply in Europe: Synthetic Cathinones 2024. Accessed August 22, 2025 (2024), https://www.euda.europa.eu/sites/default/files/pdf/31835_en.pdf?981450.

[12] M. H. Baumann , M. A. Ayestas , J. S. Partilla , et al., “The Designer Methcathinone Analogs, Mephedrone and Methylone, Are Substrates for Monoamine Transporters in Brain Tissue,” Neuropsychopharmacology 37 (2012): 1192–1203, 10.1038/npp.2011.304. 22169943 PMC3306880

[13] G. C. Hadlock , K. M. Webb , L. M. McFadden , et al., “4‐Methylmethcathinone (Mephedrone): Neuropharmacological Effects of a Designer Stimulant of Abuse,” Journal of Pharmacology and Experimental Therapeutics 339 (2011): 530–536, 10.1124/jpet.111.184119. 21810934 PMC3200001

[14] R. López‐Arnau , J. Martínez‐Clemente , D. Pubill , E. Escubedo , and J. Camarasa , “Comparative Neuropharmacology of Three Psychostimulant Cathinone Derivatives: Butylone, Mephedrone and Methylone,” British Journal of Pharmacology 167 (2012): 407–420, 10.1111/j.1476-5381.2012.01998.x. 22509960 PMC3481047

[15] J. Martínez‐Clemente , E. Escubedo , D. Pubill , and J. Camarasa , “Interaction of Mephedrone With Dopamine and Serotonin Targets in Rats,” European Neuropsychopharmacology 22 (2012): 231–236, 10.1016/j.euroneuro.2011.07.009. 21824752

[16] C. Pifl , H. Reither , and O. Hornykiewicz , “The Profile of Mephedrone on Human Monoamine Transporters Differs From 3,4‐Methylenedioxymethamphetamine Primarily by Lower Potency at the Vesicular Monoamine Transporter,” European Journal of Pharmacology 755 (2015): 119–126, 10.1016/j.ejphar.2015.03.004. 25771452

[17] S. E. Shortall , A. E. Macerola , R. T. R. Swaby , et al., “Behavioural and Neurochemical Comparison of Chronic Intermittent Cathinone, Mephedrone and MDMA Administration to the Rat,” European Neuropsychopharmacology 23 (2013): 1085–1095, 10.1016/j.euroneuro.2012.09.005. 23051939

[18] O. Wronikowska‐Denysiuk , W. Mrozek , and B. Budzyńska , “The Role of Oxytocin and Vasopressin in Drug‐Induced Reward—Implications for Social and Non‐Social Factors,” Biomolecules 13 (2023): 405, 10.3390/biom13030405. 36979340 PMC10046619

[19] T. R. Insel , “The Challenge of Translation in Social Neuroscience: A Review of Oxytocin, Vasopressin, and Affiliative Behavior,” Neuron 65 (2010): 768–779, 10.1016/j.neuron.2010.03.005. 20346754 PMC2847497

[20] R. Landgraf and I. D. Neumann , “Vasopressin and Oxytocin Release Within the Brain: A Dynamic Concept of Multiple and Variable Modes of Neuropeptide Communication,” Frontiers in Neuroendocrinology 25 (2004): 150–176, 10.1016/j.yfrne.2004.05.001. 15589267

[21] H. E. Ross and L. J. Young , “Oxytocin and the Neural Mechanisms Regulating Social Cognition and Affiliative Behavior,” Frontiers in Neuroendocrinology 30 (2009): 534–547, 10.1016/j.yfrne.2009.05.004. 19481567 PMC2748133

[22] M. Lozić , O. Šarenac , D. Murphy , and N. Japundžić‐Žigon , “Vasopressin, Central Autonomic Control and Blood Pressure Regulation,” Current Hypertension Reports 20, no. 2 (2018): 11, 10.1007/s11906-018-0811-0. 29480411

[23] H. K. Caldwell , “Oxytocin and Vasopressin: Powerful Regulators of Social Behavior,” Neuroscientist 23, no. 5 (2017): 517–528, 10.1177/1073858417708284. 28492104

[24] J. Mead and A. Parrott , “Mephedrone and MDMA: A Comparative Review,” Brain Research 1735 (2020): 146740, 10.1016/j.brainres.2020.146740. 32087112

[25] F. Pantano , R. Tittarelli , G. Mannocchi , et al., “Neurotoxicity Induced by Mephedrone: An Up‐to‐Date Review,” Current Neuropharmacology 15 (2017): 738–749, 10.2174/1570159X14666161130130718. 27908258 PMC5771050

[26] E. Papaseit , C. Pérez‐Mañá , J.‐A. Mateus , et al., “Human Pharmacology of Mephedrone in Comparison With MDMA,” Neuropsychopharmacology 41 (2016): 2704–2713, 10.1038/npp.2016.75. 27206266 PMC5026738

[27] L. Ramos , C. Hicks , R. Kevin , et al., “Acute Prosocial Effects of Oxytocin and Vasopressin When Given Alone or in Combination With 3,4‐Methylenedioxymethamphetamine in Rats: Involvement of the V1A Receptor,” Neuropsychopharmacology 38 (2013): 2249–2259, 10.1038/npp.2013.125. 23676791 PMC3773675

[28] M. R. Thompson , P. D. Callaghan , G. E. Hunt , J. L. Cornish , and I. S. McGregor , “A Role for Oxytocin and 5‐HT(1A) Receptors in the Prosocial Effects of 3,4 Methylenedioxymethamphetamine (“Ecstasy”),” Neuroscience 146 (2007): 509–514, 10.1016/j.neuroscience.2007.02.032. 17383105

[29] M. G. Kirkpatrick , S. M. Francis , R. Lee , H. de Wit , and S. Jacob , “Plasma Oxytocin Concentrations Following MDMA or Intranasal Oxytocin in Humans,” Psychoneuroendocrinology 46 (2014): 23–31, 10.1016/j.psyneuen.2014.04.006. 24882155 PMC4088952

[30] P. Georgiou , P. Zanos , J.‐A. Garcia‐Carmona , et al., “The Oxytocin Analogue Carbetocin Prevents Priming‐Induced Reinstatement of Morphine‐Seeking: Involvement of Dopaminergic, Noradrenergic and MOPr Systems,” European Neuropsychopharmacology: The Journal of the European College of Neuropsychopharmacology 25 (2015): 2459–2464, 10.1016/j.euroneuro.2015.09.015. 26475574

[31] J. M. Johns , M. S. McMurray , P. W. Joyner , et al., “Effects of Chronic and Intermittent Cocaine Treatment on Dominance, Aggression, and Oxytocin Levels in Post‐Lactational Rats,” Psychopharmacology 211 (2010): 175–185, 10.1007/s00213-010-1877-9. 20526586 PMC2910929

[32] P. Zanos , P. Georgiou , S. R. Wright , et al., “The Oxytocin Analogue Carbetocin Prevents Emotional Impairment and Stress‐Induced Reinstatement of Opioid‐Seeking in Morphine‐Abstinent Mice,” Neuropsychopharmacology 39 (2014): 855–865, 10.1038/npp.2013.285. 24129263 PMC3924520

[33] J. H. Broadbear , B. Tunstall , and K. Beringer , “Examining the Role of Oxytocin in the Interoceptive Effects of 3,4‐Methylenedioxymethamphetamine (MDMA, ‘Ecstasy’) Using a Drug Discrimination Paradigm in the Rat,” Addiction Biology 16 (2011): 202–214, 10.1111/j.1369-1600.2010.00267.x. 21070509

[34] M. Ilic , M. Holy , K. Jaentsch , et al., “Cell‐Based Radiotracer Binding and Uptake Inhibition Assays: A Comparison of In Vitro Methods to Assess the Potency of Drugs That Target Monoamine Transporters,” Frontiers in Pharmacology 11 (2020): 673, 10.3389/fphar.2020.00673. 32508638 PMC7248194

[35] A. Bahi , “The Oxytocin Receptor Impairs Ethanol Reward in Mice,” Physiology & Behavior 139 (2015): 321–327, 10.1016/j.physbeh.2014.11.046. 25449413

[36] M. Rae , P. Zanos , P. Georgiou , P. Chivers , A. Bailey , and R. Camarini , “Environmental Enrichment Enhances Conditioned Place Preference to Ethanol via an Oxytocinergic‐Dependent Mechanism in Male Mice,” Neuropharmacology 138 (2018): 267–274, 10.1016/j.neuropharm.2018.06.013. 29908241

[37] T. E. Hodges , A. M. Eltahir , S. Patel , R. Bredewold , A. H. Veenema , and C. M. McCormick , “Effects of Oxytocin Receptor Antagonism on Social Function and Corticosterone Release After Adolescent Social Instability in Male Rats,” Hormones and Behavior 116 (2019): 104579, 10.1016/j.yhbeh.2019.104579. 31449812

[38] S.‐Y. Lee , S.‐H. Park , C. Chung , J. J. Kim , S.‐Y. Choi , and J.‐S. Han , “Oxytocin Protects Hippocampal Memory and Plasticity From Uncontrollable Stress,” Scientific Reports 5 (2015): 18540, 10.1038/srep18540. 26688325 PMC4685249

[39] P. K. Olszewski , J. R. Waas , L. L. Brooks , F. Herisson , and A. S. Levine , “Oxytocin Receptor Blockade Reduces Acquisition but Not Retrieval of Taste Aversion and Blunts Responsiveness of Amygdala Neurons to an Aversive Stimulus,” Peptides 50 (2013): 36–41, 10.1016/j.peptides.2013.09.008. 24063812

[40] O. Tan , H. Musullulu , J. S. Raymond , B. Wilson , M. Langguth , and M. T. Bowen , “Oxytocin and Vasopressin Inhibit Hyper‐Aggressive Behaviour in Socially Isolated Mice,” Neuropharmacology 156 (2019): 107573, 10.1016/j.neuropharm.2019.03.016. 30885607

[41] N. A. Everett , I. S. McGregor , S. J. Baracz , and J. L. Cornish , “The Role of the Vasopressin V1A Receptor in Oxytocin Modulation of Methamphetamine Primed Reinstatement,” Neuropharmacology 133 (2018): 1–11, 10.1016/j.neuropharm.2017.12.036. 29353054

[42] O. Wronikowska , M. Zykubek , A. Michalak , et al., “Insight Into Glutamatergic Involvement in Rewarding Effects of Mephedrone in Rats: In Vivo and Ex Vivo Study,” Molecular Neurobiology 58 (2021): 4413–4424, 10.1007/s12035-021-02404-y. 34021482 PMC8487417

[43] O. Wronikowska‐Denysiuk , A. Michalak , A. Pankowska , et al., “Relationship Between GABA‐Ergic System and the Expression of Mephedrone‐Induced Reward in Rats‐Behavioral, Chromatographic and In Vivo Imaging Study,” International Journal of Molecular Sciences 24 (2023): 9958, 10.3390/ijms24129958. 37373105 PMC10298564

[44] E. L. MacLean , L. R. Gesquiere , N. Gee , K. Levy , W. L. Martin , and C. S. Carter , “Validation of Salivary Oxytocin and Vasopressin as Biomarkers in Domestic Dogs,” Journal of Neuroscience Methods 293 (2018): 67–76, 10.1016/j.jneumeth.2017.08.033. 28865986

[45] J. A. Marusich , E. A. Gay , and B. E. Blough , “Analysis of Neurotransmitter Levels in Addiction‐Related Brain Regions During Synthetic Cathinone Self‐Administration in Male Sprague‐Dawley Rats,” Psychopharmacology 236 (2019): 903–914, 10.1007/s00213-018-5011-8. 30191259 PMC6401347

[46] J. A. Marusich , E. A. Gay , S. L. Watson , and B. E. Blough , “Synthetic Cathinone Self‐Administration in Female Rats Modulates Neurotransmitter Levels in Addiction‐Related Brain Regions,” Behavioural Brain Research 376 (2019): 112211, 10.1016/j.bbr.2019.112211. 31493431 PMC6783379

[47] K. Kamińska , K. Noworyta‐Sokołowska , A. Górska , et al., “The Effects of Exposure to Mephedrone During Adolescence on Brain Neurotransmission and Neurotoxicity in Adult Rats,” Neurotoxicity Research 34 (2018): 525–537, 10.1007/s12640-018-9908-0. 29713996 PMC6154178

[48] M. Angoa‐Pérez , M. J. Kane , N. Herrera‐Mundo , D. M. Francescutti , and D. M. Kuhn , “Effects of Combined Treatment With Mephedrone and Methamphetamine or 3,4‐Methylenedioxymethamphetamine on Serotonin Nerve Endings of the Hippocampus,” Life Sciences 97 (2014): 31–36, 10.1016/j.lfs.2013.07.015. 23892197 PMC3858458

[49] P. B. Pail , K. M. Costa , C. E. Leite , and M. M. Campos , “Comparative Pharmacological Evaluation of the Cathinone Derivatives, Mephedrone and Methedrone, in Mice,” Neurotoxicology 50 (2015): 71–80, 10.1016/j.neuro.2015.08.004. 26254738

[50] C. P. Motbey , E. Karanges , K. M. Li , et al., “Mephedrone in Adolescent Rats: Residual Memory Impairment and Acute but Not Lasting 5‐HT Depletion,” PLoS One 7 (2012): e45473, 10.1371/journal.pone.0045473. 23029034 PMC3445542

[51] G. J. H. Dumont , F. C. G. J. Sweep , R. van der Steen , et al., “Increased Oxytocin Concentrations and Prosocial Feelings in Humans After Ecstasy (3,4‐Methylenedioxymethamphetamine) Administration,” Social Neuroscience 4 (2009): 359–366, 10.1080/17470910802649470. 19562632

[52] K. Wolff , E. M. Tsapakis , A. R. Winstock , et al., “Vasopressin and Oxytocin Secretion in Response to the Consumption of Ecstasy in a Clubbing Population,” Journal of Psychopharmacology (Thousand Oaks, CA) 20 (2006): 400–410, 10.1177/0269881106061514. 16574714

[53] K. M. Dumais and A. H. Veenema , “Vasopressin and Oxytocin Receptor Systems in the Brain: Sex Differences and Sex‐Specific Regulation of Social Behavior,” Frontiers in Neuroendocrinology 40 (2016): 1–23, 10.1016/j.yfrne.2015.04.003. 25951955 PMC4633405

[54] N. D. Volkow , M. Michaelides , and R. Baler , “The Neuroscience of Drug Reward and Addiction,” Physiological Reviews 99 (2019): 2115–2140, 10.1152/physrev.00014.2018. 31507244 PMC6890985

[55] G. Dölen , A. Darvishzadeh , K. W. Huang , and R. C. Malenka , “Social Reward Requires Coordinated Activity of Nucleus Accumbens Oxytocin and Serotonin,” Nature 501 (2013): 179–184, 10.1038/nature12518. 24025838 PMC4091761

[56] A. V. Williams , N. Duque‐Wilckens , S. Ramos‐Maciel , et al., “Social Approach and Social Vigilance Are Differentially Regulated by Oxytocin Receptors in the Nucleus Accumbens,” Neuropsychopharmacology 45 (2020): 1423–1430, 10.1038/s41386-020-0657-4. 32198453 PMC7360746

[57] N. I. Cilz , A. Cymerblit‐Sabba , and W. S. Young , “Oxytocin and Vasopressin in the Rodent Hippocampus,” Genes, Brain, and Behavior 18 (2019): e12535, 10.1111/gbb.12535. 30378258

[58] M. G. Kutlu and T. J. Gould , “Effects of Drugs of Abuse on Hippocampal Plasticity and Hippocampus‐Dependent Learning and Memory: Contributions to Development and Maintenance of Addiction,” Learning & Memory 23 (2016): 515–533, 10.1101/lm.042192.116. 27634143 PMC5026208

[59] T. Raam , K. M. McAvoy , A. Besnard , A. H. Veenema , and A. Sahay , “Hippocampal Oxytocin Receptors Are Necessary for Discrimination of Social Stimuli,” Nature Communications 8 (2017): 2001, 10.1038/s41467-017-02173-0. PMC5722862 29222469

[60] I. S. McGregor , P. D. Callaghan , and G. E. Hunt , “From Ultrasocial to Antisocial: A Role for Oxytocin in the Acute Reinforcing Effects and Long‐Term Adverse Consequences of Drug Use?,” British Journal of Pharmacology 154 (2008): 358–368, 10.1038/bjp.2008.132. 18475254 PMC2442436

[61] S. J. Baracz , L. M. Parker , A. S. Suraev , et al., “Chronic Methamphetamine Self‐Administration Dysregulates Oxytocin Plasma Levels and Oxytocin Receptor Fibre Density in the Nucleus Accumbens Core and Subthalamic Nucleus of the Rat,” Journal of Neuroendocrinology 28, no. 4 (2016): e12337, 10.1111/jne.12337. 26563756

[62] I. F. Bielsky , S.‐B. Hu , K. L. Szegda , H. Westphal , and L. J. Young , “Profound Impairment in Social Recognition and Reduction in Anxiety‐Like Behavior in Vasopressin V1a Receptor Knockout Mice,” Neuropsychopharmacology 29 (2004): 483–493, 10.1038/sj.npp.1300360. 14647484

[63] C. Yang , X. Zhang , J. Gao , M. Wang , and Z. Yang , “Arginine Vasopressin Ameliorates Spatial Learning Impairments in Chronic Cerebral Hypoperfusion via V1a Receptor and Autophagy Signaling Partially,” Translational Psychiatry 7 (2017): e1174, 10.1038/tp.2017.121. 28934194 PMC5538111

[64] Y.‐J. Cheng , G.‐Y. Zan , Y.‐Z. Deng , et al., “Oxytocinergic Input From the Paraventricular Nucleus to the Nucleus Accumbens Core Modulates Methamphetamine‐Conditioned Place Preference,” Nature Communications 16 (2025): 4808, 10.1038/s41467-025-59859-z. PMC12102147 40410135

[65] D. Feifel , P. D. Shilling , and A. M. Belcher , “The Effects of Oxytocin and Its Analog, Carbetocin, on Genetic Deficits in Sensorimotor Gating,” European Neuropsychopharmacology: The Journal of the European College of Neuropsychopharmacology 22 (2012): 374–378, 10.1016/j.euroneuro.2011.09.004. 21962914 PMC4208693

[66] K. Macdonald and D. Feifel , “Helping Oxytocin Deliver: Considerations in the Development of Oxytocin‐Based Therapeutics for Brain Disorders,” Frontiers in Neuroscience 7 (2013): 35, 10.3389/fnins.2013.00035. 23508240 PMC3597931

[67] M. L. S. Bates , R. S. Hofford , M. A. Emery , P. J. Wellman , and S. Eitan , “The Role of the Vasopressin System and Dopamine D1 Receptors in the Effects of Social Housing Condition on Morphine Reward,” Drug and Alcohol Dependence 188 (2018): 113–118, 10.1016/j.drugalcdep.2018.03.021. 29772497

[68] S. Edwards , M. Guerrero , O. M. Ghoneim , E. Roberts , and G. F. Koob , “Evidence That Vasopressin V1b Receptors Mediate the Transition to Excessive Drinking in Ethanol‐Dependent Rats,” Addiction Biology 17 (2012): 76–85, 10.1111/j.1369-1600.2010.00291.x. 21309953 PMC3178679

[69] X. Qi , L. Guzhva , Y. Ji , and A. W. Bruijnzeel , “Chronic Treatment With the Vasopressin 1b Receptor Antagonist SSR149415 Prevents the Dysphoria Associated With Nicotine Withdrawal in Rats,” Behavioural Brain Research 292 (2015): 259–265, 10.1016/j.bbr.2015.06.031. 26112757 PMC4558258

[70] Y. Zhou , F. Leri , E. Cummins , M. Hoeschele , and M. J. Kreek , “Involvement of Arginine Vasopressin and V1b Receptor in Heroin Withdrawal and Heroin Seeking Precipitated by Stress and by Heroin,” Neuropsychopharmacology 33 (2008): 226–236, 10.1038/sj.npp.1301419. 17443128

[71] Y. Zhou , Y. Litvin , A. P. Piras , D. W. Pfaff , and M. J. Kreek , “Persistent Increase in Hypothalamic Arginine Vasopressin Gene Expression During Protracted Withdrawal From Chronic Escalating‐Dose Cocaine in Rodents,” Neuropsychopharmacology 36 (2011): 2062–2075, 10.1038/npp.2011.97. 21677651 PMC3158323

[72] Y. Zhou , M. Rubinstein , M. J. Low , and M. J. Kreek , “V1b Receptor Antagonist SSR149415 and Naltrexone Synergistically Decrease Excessive Alcohol Drinking in Male and Female Mice,” Alcoholism, Clinical and Experimental Research 42 (2018): 195–205, 10.1111/acer.13544. 29105118 PMC5750120

[73] C. S. Gabor , A. Phan , A. E. Clipperton‐Allen , M. Kavaliers , and E. Choleris , “Interplay of Oxytocin, Vasopressin, and Sex Hormones in the Regulation of Social Recognition,” Behavioral Neuroscience 126 (2012): 97–109, 10.1037/a0026464. 22141469

